# Altered immune phenotype and DNA methylation in panic disorder

**DOI:** 10.1186/s13148-020-00972-9

**Published:** 2020-11-18

**Authors:** Curtis L. Petersen, Ji-Qing Chen, Lucas A. Salas, Brock C. Christensen

**Affiliations:** 1 The Dartmouth Institute for Health Policy and Clinical Practice, Lebanon, NH 03766 USA; 2 Quantitative Biomedical Science Program, Geisel School of Medicine at Dartmouth, Lebanon, NH 03766 USA; 3 Program for Experimental and Molecular Medicine, Geisel School of Medicine at Dartmouth, Lebanon, NH 03766 USA; 4 Department of Epidemiology, Geisel School of Medicine at Dartmouth, Lebanon, NH 03766 USA; 5 Department of Molecular and Systems Biology, Geisel School of Medicine at Dartmouth, Lebanon, NH 03766 USA; 6 Dartmouth Hitchcock Medical Center, 1 Medical Center Dr, 660 Williamson Translation Research Building, Lebanon, NH 03756 USA

**Keywords:** Panic disorder, DNA methylation, Immune system, Cell-type deconvolution, Repetitive DNA elements

## Abstract

**Background:**

Multiple studies have related psychiatric disorders and immune alterations. Panic disorder (PD) has been linked with changes in leukocytes distributions in several small studies using different methods for immune characterization. Additionally, alterations in the methylation of repetitive DNA elements, such as LINE-1, have been associated with mental disorders. Here, we use peripheral blood DNA methylation data from two studies and an updated DNA methylation deconvolution library to investigate the relation of leukocyte proportions and methylation status of repetitive elements in 133 patients with panic disorder compared with 118 controls.

**Methods and results:**

We used DNA methylation data to deconvolute leukocyte cell-type proportions and to infer LINE-1 element methylation comparing PD cases and controls. We also identified differentially methylated CpGs associated with PD using an epigenome-wide association study approach (EWAS), with models adjusting for sex, age, and cell-type proportions. Individuals with PD had a lower proportion of CD8T cells (OR: 0.86, 95% CI: 0.78–0.96, *P*-adj = 0.030) when adjusting for age, sex, and study compared with controls. Also, PD cases had significantly lower LINE-1 repetitive element methylation than controls (*P* < 0.001). The EWAS identified 61 differentially methylated CpGs (58 hypo- and 3 hypermethylated) in PD (Bonferroni adjusted *P* < 1.33 × 10^–7^).

**Conclusions:**

These results suggest that those with panic disorder have changes to their immune system and dysregulation of repeat elements relative to controls.

## Background

Panic disorder (PD), an anxiety disorder, is characterized by sudden and repeated, unexpected panic attacks. Panic attacks usually last ten minutes and are defined as A: palpitations or racing heart, sweating, shortness of breath, chest and stomach pain, feeling dizzy and shaking or trembling B: fear of dying, choking feeling, feeling unsteady or faint, feeling unreal, fear of being out of control and worrying about the next panic attack [1]. According to the survey of National Institute of Mental Health (NIMH) and National Comorbidity Survey Replication (NCS-R), an estimated 2.7% of adults (age > 18) had a panic disorder in the U.S. (2001–2003), and 4.7% of U.S. adults had ever experienced panic disorder [2]. Additionally, the lifetime prevalence of PD is higher for women (7.1%) than for men (4.0%) [3], and its prevalence changes as individuals age—2.8% in 18–29 years old, 3.7% in 30–44 years old, 3.1% in 45–59 years old, and 0.8% in those 60 + years old [2]. Beyond sex and age, panic disorder has also been associated with other comorbidities like agoraphobia, clinical depression [2], hypertension [4], diabetes [5], and irritable bowel syndrome, along with higher utilization of health systems [6]. Together, these findings suggest a complex mechanism that is impacted through experience, environment, and biology.

New approaches to understand and investigate molecular and physiological mechanisms of mental health conditions are needed, and epigenetic marks are currently being investigated in PD. DNA methylation is the covalent addition of a methyl group to a cytosine, usually in the context of CpG dinucleotides that serves to regulate gene expression and cell lineage specification. The combination of reference DNA methylation profiles from purified immune cell types and statistical techniques allows peripheral blood DNA methylation measures to be used to infer immune cell proportions [7, 8]. This DNA-based approach has the advantage of being amenable to archival blood samples, unlike traditional cell sorting methods. Previously, in efforts to study PD's relation with immune status, researchers employed immunostaining and flow cytometry to measure numbers and proportion of lymphocytes. Here, we used publicly available genome-scale peripheral blood DNA methylation data from two independent studies of panic disorder cases and controls to infer immune cell proportions [9, 10], and test the relation of leukocyte subtype proportions with panic disorder case status comparing to healthy controls. In addition, to assess potential immunosuppression in PD cases versus controls, we calculated the methylation-derived neutrophil-to-lymphocyte ratio (mdNLR) [11]. Due to the differential association that sex and age have with PD [2, 3], and immune status [12], we also tested the association between PD and leukocyte proportions and mdNLR in adjusted models. Finally, using genome-wide methylation data allowed us to infer methylation of repeat elements, which make up a large proportion of the human genome and have been demonstrated to have differential methylation in neurological and psychiatric conditions [13–15].

Although reports have shown evidence of differing immune phenotypes for patients with panic disorder, the results of these studies have been inconsistent and often only include small numbers of subjects. Increased B lymphocyte and NK cell proportions with concomitantly decreased T cell proportions have been observed in PD patients compared with controls (n = 41 and n = 52) [16, 17]. However, other work has observed lower absolute counts of B lymphocytes and no difference in other cell-type proportions (n = 28) [18]. Lower proportions of CD8 T cells have also been observed in PD patients (n = 40) [19], and a study investigating the impact that PD had on immune functioning found that it induced higher natural killer cell activity than those without PD (n = 28) [20]. Finally, three recent studies have examined the potential role of DNA methylation in PD risk. Two studies measured DNA methylation in peripheral blood with the 450 K array in PD patients. Iurato et al. [9] recruited PD patients (n = 89) and controls (n = 76) through the Max Planck Institute of Psychiatry (MPIP) in Munich, Germany. Shimada-Sugimoto et al. [10] recruited individuals with PD (n = 48) and controls (n = 48) living in Tokyo and Nagoya, Japan. The third study recruited 57 PD patients and 61 controls at the University of Wuerzburg, Germany, and measured peripheral blood DNA methylation with the MethylationEPIC array, though data were not archived or available upon request at the time of our analysis [21].

While the original studies adjusted for cell-type heterogeneity in epigenome-wide analyses, Shimada-Sugimoto et al. [10] tested the relation of cell-type proportion with PD case status using the Reinius library from 2012 [7], which was based on six healthy Swedish males 25–60 years old. To expand on the work of Iurato et al. [9] and Shimada-Sugimoto et al. [10] (in a combined analysis) of immune phenotype and its relationship with the panic disorder, we use the updated cell-type reference data from Salas et al. [8] that includes ethnically diverse subjects, both men and women, and has improved deconvolution accuracy compared with the Reinius library.

## Results

**Fig. 1:**
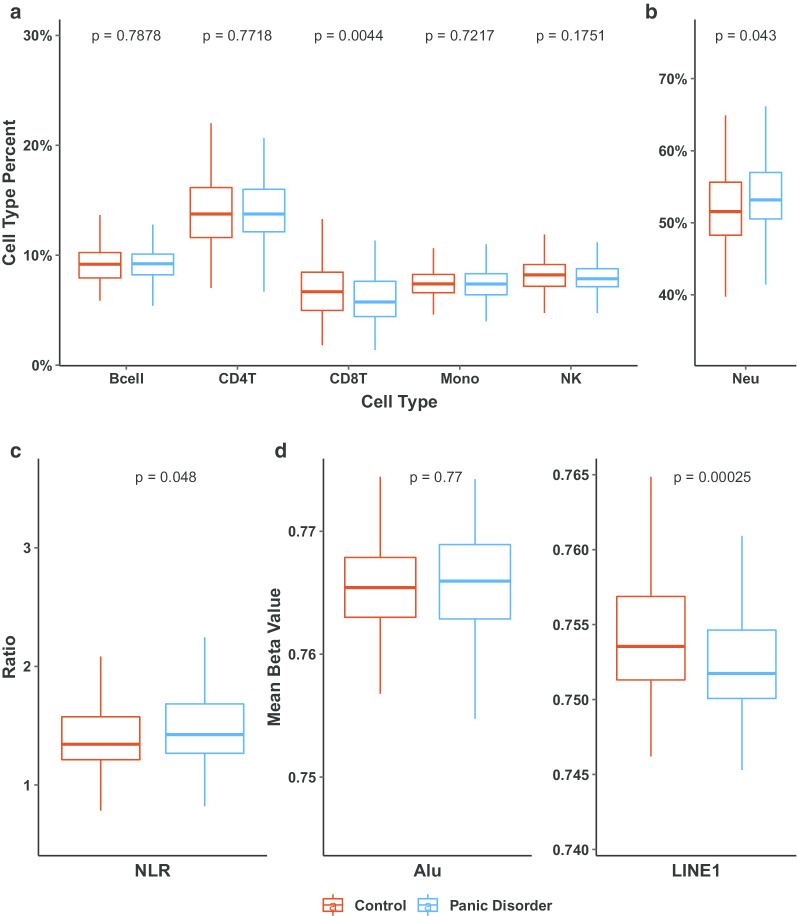
Comparison of blood cell proportions and methylation-derived neutrophil-to-lymphocyte ratio (mdNLR), in Panic Disorder cases and controls. **a** Leukocyte proportions—only CD8T cells showed a significant difference between PD patients compared to controls. **b** Neutrophil percent was significantly larger in PD patients compared to controls. **c** mdNLR was significantly higher in PD patients relative to controls. **d** No difference was observed in Alu repeat elements while there was significant LINE-1 hypomethylation in those with panic disorder

The 251 total participants were 60.6% women (n = 152) had a mean Horvath methylation derived age of 38.8 years (10.2 SD), with 133 (53%) participants diagnosed with panic disorder (PD). Details of study population characteristics were described in each original study and are also provided in Table 1 [9, 10]. In the combined analysis of immune cell proportions in cases and controls from both studies, we observed significantly lower proportions of CD8+ T-lymphocytes in PD cases compared with controls (− 0.97%, *P* = 0.0044, Fig. 1a). In addition, we observed significantly higher neutrophil proportions in PD cases compared with controls (1.20%, *P* = 0.043, Fig. 1b). The methylation-derived neutrophil-to-lymphocyte ratio (mdNLR) was also significantly elevated in PD cases compared with controls (0.06%, *P* = 0.048, Fig. 1c). Summary statistics of cell-type proportions and mdNLR estimates among all subjects and stratified by case status are shown in Table 2. In models adjusted for age and sex, there was a reduction in CD8+ T-lymphocytes and (OR: 0.86, 95% CI: 0.78–0.96, *P*-adj = 0.030), increased mdNLR was still evident in PD cases, though it did not reach statistical significance (OR: 1.53, 95% CI: 0.81–3.00, *P*-adj = 0.794).

**Table 1:** Study participant characteristics

	Control (N = 118)	Panic disorder (N = 133)	Total (N = 251)
Sex, n (%)			
Male	45 (38.1)	54 (40.6)	99 (39.4)
Female	73 (61.9)	79 (59.4)	152 (60.6)
Age, mean (sd)^a^	40.0 (9.0)	37.8 (11.0)	38.8 (10.2)
Study			
Iurato et al. [9], n (%)	70 (59.3)	85 (63.9)	155 (61.8)
Shimada-Sugimoto et al. [10], n (%)	48 (40.7)	48 (36.1)	96 (38.2)

^a^Methylation derived, Horvath library

**Table 2:** Methylation-derived cell-type estimates (%)

Cell type	Control (N = 118) Mean (SD)	Panic disorder (N = 133) Mean (SD)	Total (N = 251) Mean (SD)
B-cell	9.23 (1.94)	9.25 (1.75)	9.24 (1.84)
CD4T	13.63 (3.10)	13.66 (3.14)	13.64 (3.12)
CD8T	7.37 (2.78)	6.40 (2.40)	6.86 (2.63)
Mono	7.60 (1.49)	7.50 (1.47)	7.55 (1.48)
Neu	51.92 (5.83)	53.12 (5.56)	52.55 (5.71)
NK	8.31 (1.62)	8.06 (1.47)	8.18 (1.55)
mdNLR	1.41 (0.41)	1.47 (0.38)	1.44 (0.39)

In an analysis of repeat element methylation, we observed no difference in mean methylation of short interspersed nuclear elements (Alu repeats) between PD cases and controls. However, the mean methylation of LINE-1 (Long interspersed nucleotide element-1) was significantly lower in PD cases compared with controls (difference in mean beta value = − 0.002, *P* < 0.001, Fig. 1d), and remained significant in a model adjusted for age, sex, and study, (*P* < 0.001).

One of the main objectives of Iurato et al. [9], and Shimada-Sugimoto et al. [10], was to examine the differential methylation status at loci across the genome associated with PD. Iurato et al. [9] and Shimada-Sugimoto et al. [10] found 0 and 40 significantly differentiated CpGs, respectively. Since these studies have been published, more accurate cell-type estimation methods have emerged. Here, combining data from both studies, we tested the relation of methylation with panic disorder case status epigenome-wide (EWAS) using models adjusted for age, sex, and cell-type proportions. In the EWAS combining data from both studies, we identified 61 significantly differentially methylated CpGs associated with PD (Bonferroni adjusted *P* < 1.33 × 10^–7^, Additional file 1 and Additional file 2: Figure 1). We observed a trend of significant hypomethylation in PD cases with 58 of 61 sites hypomethylated, and the trend was consistent among the 1,560 CpGs at FDR-Q < 0.05 (Additional file 2: Figure 2). Sensitivity analyses to explore the potential contribution of other unobserved confounders or smoking exposure were also performed. To attempt to adjust for unobserved confounders, we used the OSCA MOMENT method, which produced similarly ranked CpGs in association with PD, though with somewhat attenuated *P* values (Additional file 2: Figure 3). Models including smoking status predicted with EpiSmokEr [22], showed highly consistent results with our EWAS where 42 of 61 CpGs identified at Bonferroni adjusted *P* 1.33 × 10^–7^, and 1,340 of 1,347 CpGs (99.5%), with FDR-Q < 0.05 among the 1,560 CpGs above.

**Fig. 2:**
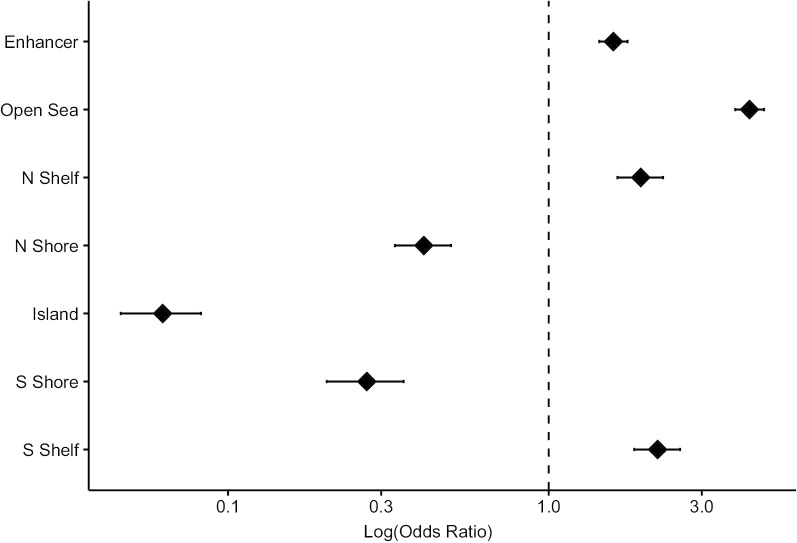
Genomic context enrichment analysis of CpG sites whose methylation state is significantly associated with panic disorder. For the 1560 CpG sites from our EWAS, we stratified by genomic context and tested for enrichment versus all modeled CpG sites. Each point is the log odds ratio for CpGs associated with panic disorder in each region, and the bar represents the 95% CI. Values greater than 1 indicate enrichment values less than 1 indicate depletion of genomic context among PD associated CpG sites

With a strong trend of differential hypomethylation in PD cases, we next explored the genomic context distribution CpGs. Here, to encompass additional CpGs for enrichment testing we expanded the CpG set beyond those meeting the Bonferroni *P* value cutoff to include those at FDR-Q < 0.05. Examining the genomic context of the 1,560 PD-associated CpGs, we observed that there was a significant enrichment of CpGs that tracked to the enhancer, open sea, and CpG island shelf regions. PD-associated CpGs were significantly depleted in CpG islands and CpG island shore regions (Fig. 2). Locus Overlap Analysis (LOLA) [23] was used to examine potential enrichment of the 1560 CpGs differentially associated with PD in known genomic contexts. We observed several significant enrichments (Q < 0.01) for CpG in several types of genomic regions. Of the UCSC defined genomic region types [24], repeats—RepeatMasker [25] and nested repeats—were enriched for CpGs hypomethylated in individuals with PD relative to controls. Additionally, deletions and duplications identified in the Coriell cell lines and lamin B1-associated domains (LADs) were also significantly enriched for CpGs hypomethylated in individuals with PD relative to controls (Additional file 2: Figure 4). Using the Gene Set Enrichment Analysis (GSEA) molecular signature database, we identified 12 pathways consisting of 216 genes that were significantly enriched among PD associated CpGs (adjusted *P* < 0.05, Additional file 2: Table 1). Of the CpGs identified in our linear models, 36 were linked to 11 of these pathways. When examining these overlapping CpGs, the majority of the significant CpGs were in the Wendt identified cohesin targets pathway [26].

## Discussion

Previous studies have examined differential methylation within panic disorder (PD), and one has examined differential immune functioning [9, 10, 21]; none have examined the role of the neutrophil to lymphocyte ratio. The NLR has been utilized to assess inflammatory response, and an elevated mdNLR has been associated with poor prognosis in several cancers [27, 28], along with reduced survival [29]. In this meta-analysis of two independent cohorts, we observed differential cell-type proportions in PD case peripheral blood compared with control subjects using improved methods for cell-type deconvolution.

A relationship between depressive/anxiety disorders and immune functioning has long been examined [30], exploring how inflammatory signaling pathways may contribute to the pathophysiology in neurotransmitter function, neuroendocrine function, and neural circuitry [30, 31]. Our results support the hypothesis that a similar mechanism is contributing to PD. A recent study demonstrated that inflammation-related miRNAs target axon guidance and neurotransmitters [32], suggesting, in this context, that exo-miRNAs could be involved in the dysregulation of immune cells in panic disorder while impacting neurological functioning.

In previous studies, exposure to stress or trauma in anxiety-based disorders activates a peripheral inflammatory response that leads to increased circulating concentrations of cytokines, such as IL-1β and IL-6 [33, 34]. Those with panic disorder have also demonstrated increased IL-1β and IL-6 [35, 36]. This is important in this context as IL-1β and IL-6 promote the differentiation and activation of naïve CD8+ T cells and enhance the effector function of activated CD8+ T cells [37–39]. Activated CD8+ T cells can secrete TNF-α and IFN-γ in response to inflammation, we observed lower CD8+ T cell proportions in panic disorder cases, consistent with lower levels of IFN-γ in PD cases observed in prior work [40]. Higher levels of IL-1β and IL-6 in panic disorder patients may interfere with differentiation and activation of naïve CD8+ T, possibly reducing inflammation. Links between inflammatory response and presentation of neurodegeneration and neuropsychological performance [41, 42] are consistent both with our results and the limited evidence for neuropsychological impairments in PD patients [43]. Although cause and consequence remain unclear, additional research into immune modulation for PD cases could have value for patients and provide options to treating physicians. It is critical to get the most accurate estimate of cell-type proportions through the most up-to-date methods and modeling, to examine the association of panic disorder and immune cell-type distribution.

The observed hypomethylation in LINE-1 elements in those with panic disorder is consistent with observations in individuals with bipolar disorder and schizophrenia [44]. Though we are not aware of studies examining the potential association of repeat element methylation with panic disorder, there is building evidence of associations between reduced methylation of repeat sequences (that encode transposable elements) and the pathophysiology of schizophrenia and mood disorders [14]. Hypomethylation of repeat elements can contribute to genomic instability through increased DNA damage and the potential expression of retrotransposable elements [45, 46]. The expression of retrotransposable elements can include endogenous retroviruses leading to dsRNAs that may alter gene expression or immune regulation. Though our data are in peripheral blood and it is unclear whether decreased CD8-T lymphocyte proportions are specifically a consequence of LINE-1 hypomethylation, in an analysis of 21 tumor types with nearly 7000 samples, reduced repeat element methylation was associated with reduced infiltration of CD8-T lymphocytes [47]. LINE-1 hypomethylation and activation have been associated with autoimmune diseases [49–51], further affirming the observed hypomethylation and reduction in CD8-T cell proportions.

The EWAS of panic disorder found several CpGs associated with PD when controlling for age, sex, and cell-type. Because these differences are observed in peripheral blood and not brain tissue, it may not reflect the changing neural circuitry itself. Over 90% of the significantly differentially methylated CpGs in PD cases were hypomethylated relative to controls in adjusted models. With a Bonferroni *P* < 1.33 × 10^–7^ the 5 most significant *p *values corresponded to 14 CpGs (due to tied *p* values) and 11 genes, all of which were hypomethylated in PD cases: *PBK* (cg21177558), *SHOC1* (cg01822570), *TSBP1* (cg08475898), *NDUFAF4* (cg05472743), *CD2AP* (cg02777447), *PIK3C2G* (cg05168215), *SLCO1A2* (cg11704114), *ACSM3* (cg09539395), *ERI2* (cg09539395), *SMARCA5* (cg08619385), *CFAP206* (cg22010963), *MEP1A* (cg02449202).

Of these, several stand out given previous studies. PDZ binding-kinase (*PBK*) (also named T-lymphokine-activated killer cell-originated protein kinase (*TOPK*)) has wide-ranging regulation, including cell growth and immune function [48, 49]. Solute Carrier Organic Anion Transporter Family Member 1A2 (*SLCO1A2*) has been implicated in transporting sulfated steroids across the blood–brain barrier [50]. SWI/SNF Related, Matrix Associated, Actin-Dependent Regulator Of Chromatin, Subfamily A, Member 5 (*SMARCA5*) is highly expressed in the brain and has been demonstrated to play an important role in brain development in mice [51]. It has also been found to play a role in chromatin remodeling during stress-induced depressive behavior as part of the ACF complex [52]. Together with our LINE-1 results, this suggests a change in chromatin structure.

In the EWAS analysis, the highest number of significantly differentially methylated CpGs associated with a particular gene tracked to chromodomain helicase DNA-binding 2 (*CHD2*), which had five CpGs. Of these, all three in open sea regions 200–1500 bases upstream of the transcription start site were hypomethylated in PD, while the two remaining CpGs were both hypermethylated in the north shore region of the same CpG island. Mutations in *CHD2* have been associated with seizures and abnormal brain function, and as it appears to impact only nerve cells [53] may play a role in PD patients.

Of the 3 CpGs that were found to be associated with PD here and by Shimada-Sugimoto et al. [10], two have been reported in relevant studies. (A) cg20340149 is located in the transcription start site (TSS) of *CLASP1,* which has been associated with neuron development [54]. (B) cg05910615 lies in the TSS of *HSPB6,* which has been associated with neurological disorders [55, 56]. The differences between the study-specific differentially methylated CpG sub-analysis that we identify and those originally reported can partially be attributed to the use of updated and accurate cell-type deconvolution methods.

Finally, we identified several genomic features that are enriched for CpGs associated with PD. We observe that nested repeats and RepeatMasker defined features are enriched in those with PD, which is in concordance with our observation that LINE-1 is significantly hypomethylated in those with PD. Lamin B1-associated domains (LADs) were also enriched with CpGs associated with PD. These results further suggest that the epigenetic structure of those with PD is different compared to controls. The impact that this has on gene products and pathways is less clear. Of the 12 GSEA pathways associated with PD, three stand out—cohesin targets, nuclear factor κ-light-chain-enhancer of activated B cells (NF-κB), and neurotransmitter secretion as enriched with PD associated CpGs. In conjuncture with LINE-1 results, again highlights the potential for chromatin restructure. One gene within the cohesion target set identified is activated leukocyte cell adhesion molecule (*ALCAM*), further supporting the difference in CD8T cells. *ALCAM* has also been indicated in mediating the blood–brain-barrier's permeability in a multiple sclerosis mouse model [57]. The NF-κB pathway has been indicated in regulating neuroinflammation [58–60] and can impact neurons, glia, and cerebral blood vessels in diverse ways [58]. These results are also notable, given the hypothesized neurochemical and neurobiological origin of PD and the subsequent and medication treatments target neurotransmitters. As mentioned by Ziegler et al., some have reported the correlation of methylation status between blood and brain tissue. This observation may indicate a similar state in the brain [61–63].

Because Ziegler et al. used the EPIC array to measure methylation status, it is difficult to make direct comparisons of the methylation status of CpGs with our combined analysis of 450 k based data. While cell-type proportions were not reported, one significant CpG we found in *SMYD3* (SET and *MYND* Domain Containing 3)—was also identified by Ziegler et al. in their PD to control cross-sectional study.

While we are unable to make any causal conclusions from this meta-analysis, the associations identified warrant further investigation in the pathophysiology of panic disorder. An additional limitation was the 10,247 CpGs missing data in the 333,479 CpGs, which had to be imputed. We would expect that the k-means method used would shift the results towards the null—reducing any observed effect. The use of ComBat would also reduce any observed effect size. Due to the lack of data on the study participant age, we could not examine the potential difference in DNA methylation age and chronological age, which could be examined in future studies. Other missing covariate data, such as smoking, and medication, limit the potential confounders that can be controlled for and examined. Our cell-type proportion results are not entirely consistent with previous reports [10]. While earlier studies of DNA methylation in PD used up to date approaches, more recent advances in immune cell-type methylation libraries allowed increased accuracy for cell-type inference in this study. Differences in cell-type using previous methods and those used in this study can result in meaningful differences that we could demonstrate (Additional file 2: Figure 5), suggesting that using updated cell-type proportions is warranted.

Our analysis highlights the benefits and some challenges of meta-analysis with DNA methylation data. Building on previous studies, the meta-analysis of DNA methylation data can lead to additional insights through increased power and diversified study populations. We addressed the challenge of using separate data sets through advancement in analytic methods for DNA methylation data to remove batch effects.

## Conclusions

Here, we demonstrate the utility of DNA methylation measures in peripheral blood to test immune phenotype associations with panic disorder. We identified significantly different immune phenotypes and dysregulation of repeat elements in PD compared with control subjects.

## Methods

### Study subjects and samples

The subjects and data used in this work have been previously described in their respective publications [9, 10]. Briefly, Iurato et al. [9] recruited panic disorder (PD) patients (Diagnostic and Statistical Manual of Mental Disorders, 4th Edition (DSM-IV) criteria—Additional file 2: Table 2) through the Max Planck Institute of Psychiatry (MPIP) in Munich, Germany, described elsewhere [9]. They excluded patients with PD due to a medical or neurological condition or the presence of comorbid personality disorders (axis II disorders in the DSM-IV classification). They recruited age-matched and sex-matched controls from a Munich-based community sample. Subjects did not take any mental health medication for at least four weeks before providing a blood sample. Study data were accessed through the Gene Expression Omnibus (GEO) GEO Series (GSE) number GSE102468. Raw probe intensity data (IDAT files) were accessed and downloaded directly.

Shimada-Sugimoto et al. [10] recruited Japanese individuals living in Tokyo and Nagoya. PD diagnosis was verified and defined through medical records and the Diagnostic and Statistical Manual of Mental Disorders, 4th Edition (DSM-IV) criteria [64] based on responses to the Mini-International Neuropsychiatric Interview (MINI) [65]. There was no data on mental health medication. Study data was access through the Nation Bioscience Database Center (NBDC) of the Japan Science and Technology Agency (JIST). Data set JGAS0000000011 contained preprocessed beta values for all samples.

### DNA methylation data

As previously described, peripheral blood processing, extraction of genomic DNA, and bisulfite conversion were conducted before DNA methylation measures in the original studies. In both studies, genomic DNA was bisulfite converted using the Zymo EZ-96 DNA Methylation Kit (Zymo Research), and DNA methylation levels were measured at > 480,000 CpG sites using the Illumina HumanMethylation450 BeadChip array [9, 10]. Hybridization and processing were performed according to the instructions of the manufacturer.

Probe intensity data (IDAT files) from Iurato et al. [9] study were imported into R version 3.5 [66]. Data were processed with the minfi [67] version 1.24.0 (https://bioconductor.org/packages/minfi/) R package. Ten samples were removed due to more than 5% of their probes’ signal intensity failing to be significantly higher than background (low signal intensity; mean negative detection *p* value > 0.05), leaving 155 samples in the analysis. Additionally, 26,974 CpGs were removed from for analysis due to poor performance across samples (mean negative detection *p* value > 0.05). Beta values were quantile-normalized and combined with the beta values from Shimada-Sugimoto et al. [10]. Beta values from Shimada-Sugimoto et al. [10] were imported into R version 3.5 and 10,247 partially missing probes were imputed through k nearest neighbors [68]. Of the 485,512 probes in the Shimada-Sugimoto et al. [10] dataset, 2,065 were removed from for analysis due to poor performance (mean negative detection *p* value > 0.05). The combined beta values were limited to the 376,602 CpGs that were in both data sets and normalized along with the reference betas through beta-mixture quantile (BMIQ) [69] normalization method, and a detected batch effect was addressed through the use of ComBat [70].

### Statistical analysis

Cell-type proportions were determined for each sample through a modified version of the Houseman method [71] using the *projectCellType_CP* function [8] from the FlowSorted.Blood.EPIC package in Bioconductor. Due to missing data for CpGs in standard deconvolution DMR libraries, we generated a library from available data. The reference betas were determined through the meffil.celltype.specific.methylation [72] function resulting in 1,629 unique CpGs contained in the combined data set. To verify the efficacy of the identified CpGs, we used them to estimate cell-type proportions of samples with known proportions (Additional file 3). The resulting R^2^ for each cell type ranged from 0.915 to 0.999 (Additional file 2: Figure 6). As age was not available at the subject level in covariate data, it was estimated through the watermelon [73] implementation of the Horvath age estimation [74] and OSCA method [75, 76]. Comparing the distribution of these age estimates to the originally published study mean by Iurato et al. [9] Horvath-based age estimates were used in subsequent modeling (Additional file 2: Figure 7). mdNLR was calculated through grouping leukocyte subtypes as previously established [11]. Differences in cell-type proportions between individuals with Panic Disorder and Controls were assessed using the Wilcoxon signed-rank test. To determine if there were differences between cell-type proportions using these updated methods and those previously used, we calculated cell-type proportions using minfi as reported and tested the mean difference between each (Additional file 2: Figure 5). Unconditional multivariable logistic regression models were fit to test the association of CD8-T and mdNLR with PD case status while controlling for age and sex while allowing for each study to have a random effect [77, 78]. We used the Holm–Bonferroni method to adjust for multiple testing reported as *P*-adj. Methylation status of repeat elements, Alu, and long interspersed nucleotide element-1 (LINE-1), was estimated through the use of REMP R-package [79]. The mean beta of all Alu and LINE-1 elements was taken separately for each individual. The difference between those with and without panic disorder was compared using the Wilcoxon signed-rank test. Unconditional multivariable logistic regression models were again fit to test the association of Alu and LINE-1 with PD case status while controlling for age and sex while allowing for each study to have a random effect.

### Epigenome wide association study (EWAS)

To examine the association of specific methylated CpGs with PD diagnosis, we performed a combined one-stage individual participant data meta-analysis and study-specific analysis. We fit linear models for each CpG controlling for age, sex, immune cell proportions, and a random-effect of study (combined model only) [80]. We defined significance as a Bonferroni threshold of 1.33e−7 for the 450 k array. We relaxed this assumption for enrichment analyses using a more lenient false discovery rate (FDR) q-value of < 0.05. The same model structure and specifications were used with Alu and LINE-1 methylation status to examine the association of specific methylated non-LTR retrotransposons with a PD diagnosis.

In a sensitivity analysis, we examine potential unmeasured confounders with the OSCA MOMENT method [75]. To test if smoking may be a confounder, we first predicted smoking status for each individual through EpiSmokEr [22]. We then added smoking status to an additional limma model with age, sex, immune cell proportions, and a random-effect of study.

### Enrichment analysis

Departing from the list of CpGs, whose methylation status was associated with PD, we used three methods to examine if they were enriched in genomic features and biological pathways. First, we tested if PD associated CpGs were enriched by CpG island-related genome context through independent logistic regression models for being in enhancers, open sea, north shelves, north shores, islands, south shores, or south shelves. Second, to determine the enrichment of genomic features, we used Locus Overlap Analysis (LOLA) software [23], limiting to UCSC-defined features. Finally, we used an empirical Bayes method for pathway and gene enrichment through the ChAMP R package [81] linked to the Gene Set Enrichment Analysis (GSEA) molecular signature database.

## Supplementary information

**Additional file 1**. Epigenome-wide association study CpGs associated with Panic Disorder adjusted for age, sex, and cell-type proportions.

**Additional file 2**. Supplemental tables and figures.

**Additional file 3**. CpGs and weights used in cell-type proportion deconvolution.

## Data Availability

The dataset generated by Iurato et al. [9] analyzed during this current study is available in the gene expression omnibus repository at GSE102468. The dataset generated by Shimada-Sugimoto et al. [10] analyzed during this study is available in the gene expression omnibus repository at the National Bioscience Database Center NBDC (https://biosciencedbc.jp/en/).

## Abbreviations

PD: Panic disorder
CpG: Cytosine–guanine
mdNLR: Methylation-derived neutrophil-to-lymphocyte ratio
EWAS: Epigenome-wide association study
OR: Odds ratio
SD: Standard deviation
LADs: Lamin B1-associated domains
NIMH: National Institute of Mental Health
NCS-R: National Comorbidity Survey Replication
LINE-1: Long interspersed nucleotide element-1
LOLA: Locus Overlap Analysis
GSEA: Gene Set Enrichment Analysis
DSM-IV: Diagnostic and Statistical Manual of Mental Disorders, 4th Edition
MINI: Mini-International Neuropsychiatric Interview
MPIP: Max Planck Institute of Psychiatry
NBDC: National Bioscience Database Center
JIST: Japan Science and Technology Agency
BMIQ: Beta-mixture quantile
FDR: False discovery rate
TSS: Transcription start site
