# Supplementary material for: Altered immune phenotype and DNA methylation in panic disorder
Source: Clin Epigenetics. 2020 Nov 18;12:177. doi: 10.1186/s13148-020-00972-9 (PMC7672933; doi:10.1186/s13148-020-00972-9)
Supplement: Supplementary file 2 — Additional file 2. Supplemental tables and figures. [file 13148_2020_972_MOESM2_ESM.docx]

**Supplemental Material**

**Supplemental Figure 1:** Manhattan plot of CpGs significantly associated with PD when controlled for age, and sex, and cell-type. Combined data sets in a mixed-effects model clustered as the study level (A), data from Iurato et al. (2017) alone (B), data from Shimada-Sugimoto et al. (2017) alone (C).

A.


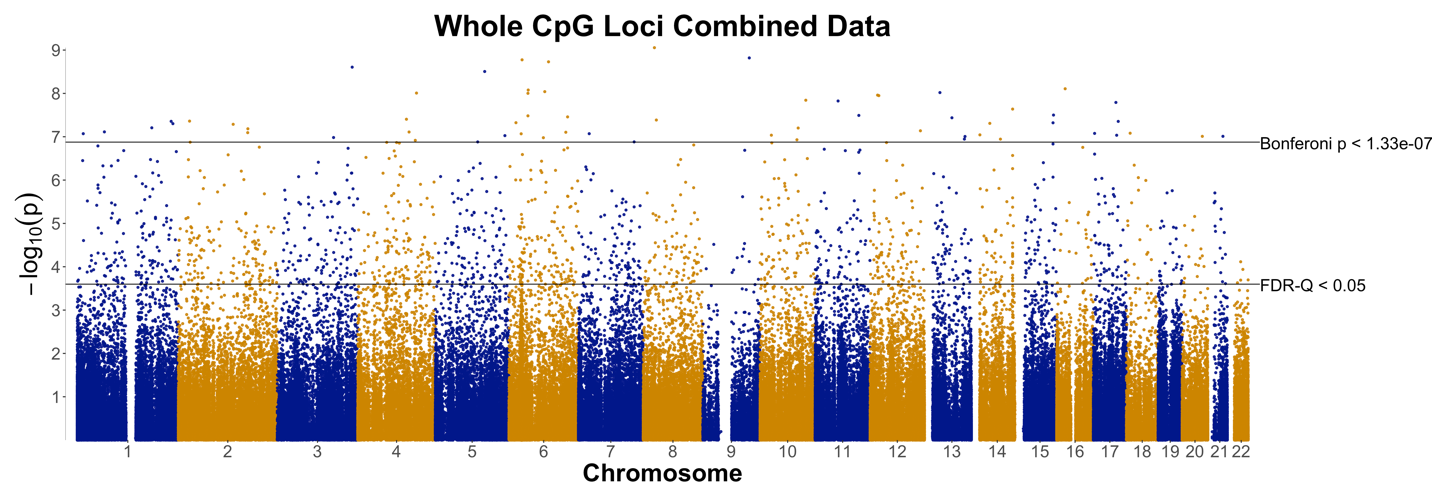


B
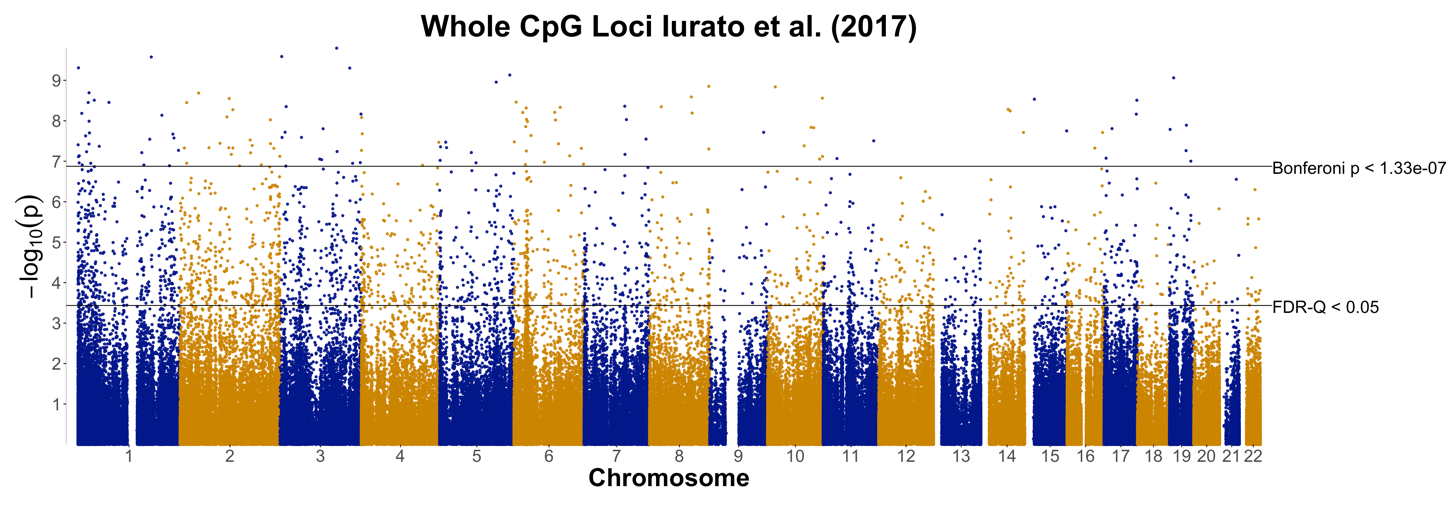


C.
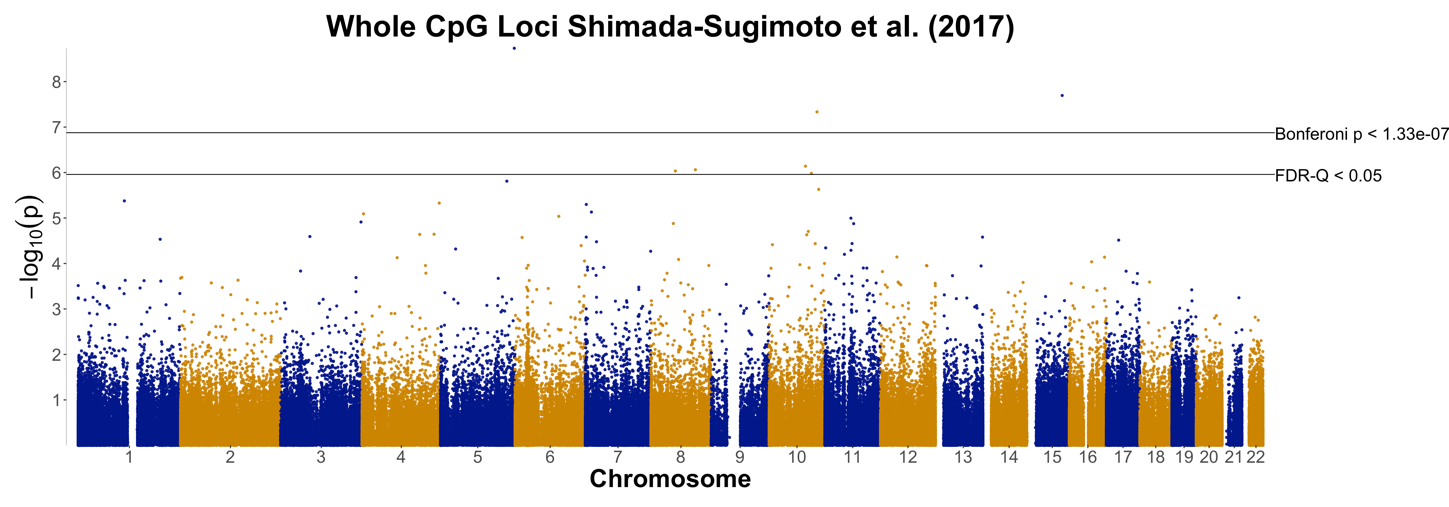
**Supplemental Figure 2:** Volcano plot of differential methylation results from the combined study EWAS for panic disorder from age, sex, and cell-type adjusted models. A total of 61 CpGs were associated (Bonferroni adjusted P < 1.33x10^-7^) with PD, 58 CpGs were hypomethylated and 3 were hypermethylated in PD cases relative to controls. Using an FDR Q < 0.05 signficance cutoff 1,560 CpGs were associated, 1,409 hypomethylation, and 151 hypermethylation in PD cases compared with controls.

**
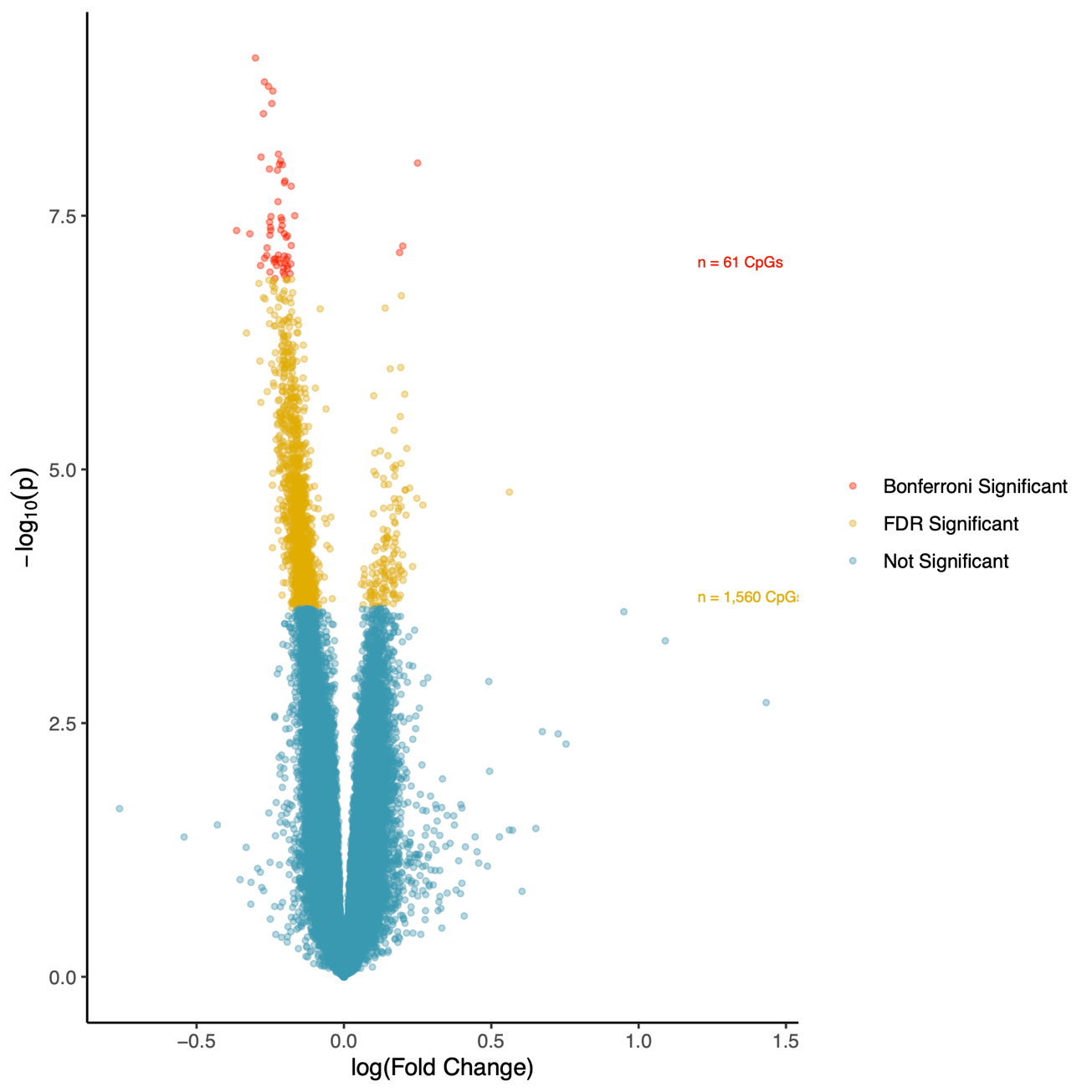
**

**Supplemental Figure 3**: Manhattan plot of CpGs significantly associated with PD identified (p≤ 0.05) through OSCA MOMENT method.


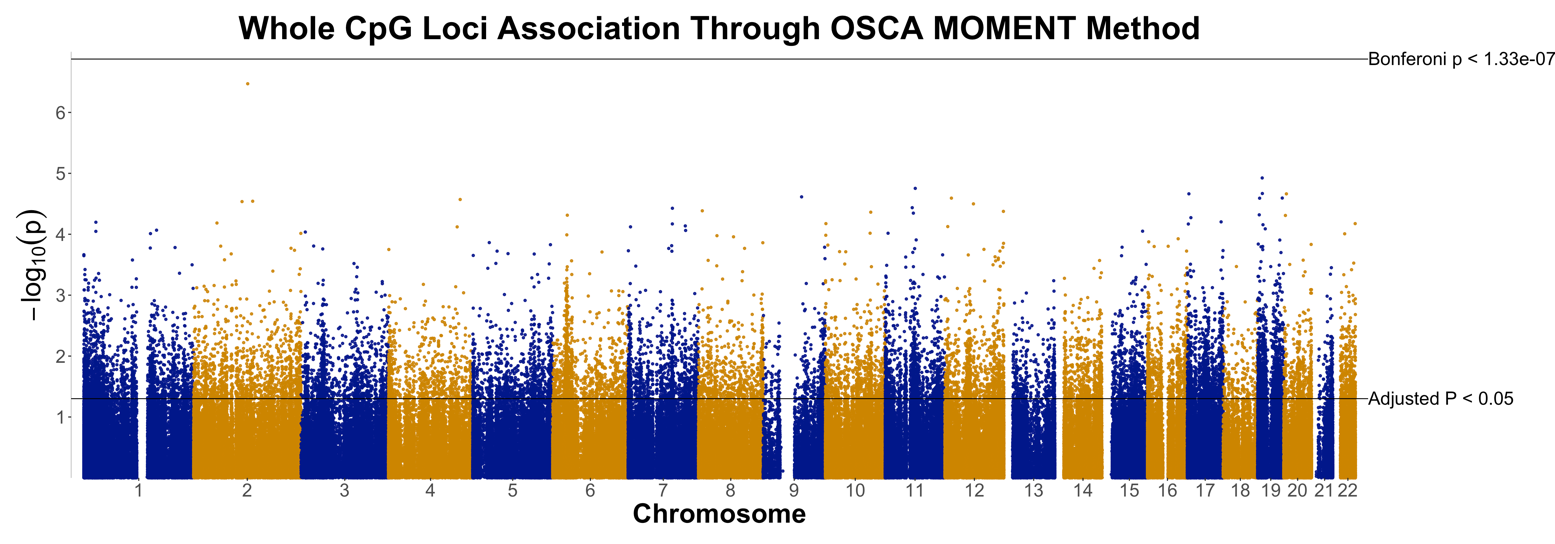


**Supplemental Figure 4:** Enrichment of CpGs associated with PD from EWAS at genomic features using locus overlap analysis (LOLA). ReapeatMasker (OR = 2.0, Q value = 9.4e-32), nested repeats, (OR = 1-9, Q value = 9.5e-18), lamina B1 associated domains (LADs) (OR = 1.7, Q value = 7.2e-20), and Coriell cell line identified deletions and duplications (OR = 1.2, Q value = 0.0064), were enriched with significantly differentially methylated CpGs in those with PD compared to controls.


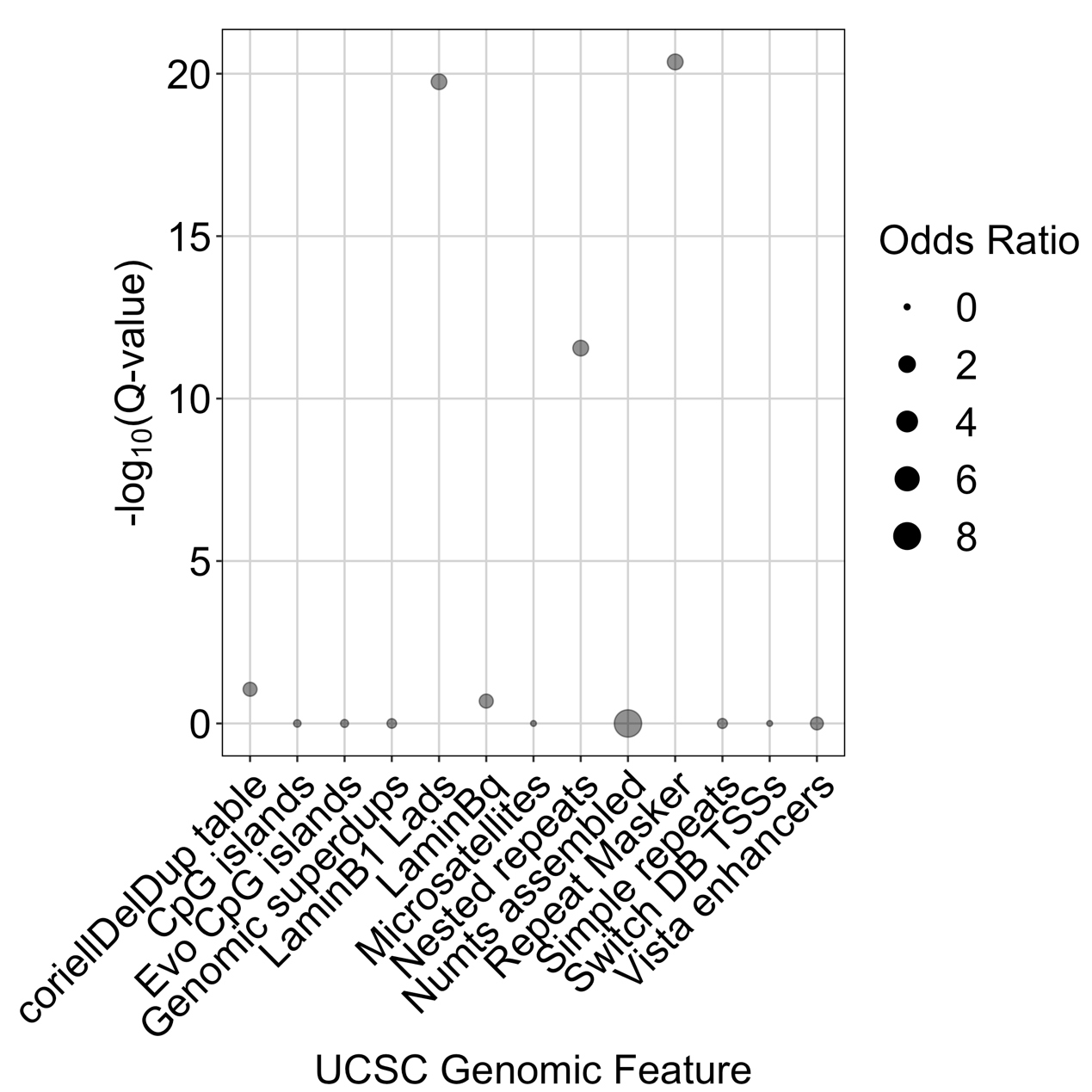


**Supplemental Table 1:** Enrichment of PD associated CpGs into Gene Set Enrichment Analysis (GSEA) defined pathways. Pathways are limited to those with an AUC of at least 0.75 and an adjusted p-value of ≤ 0.05.

| **Gene Set** | **AUC** | **Adjusted p-value** |
| --- | --- | --- |
| Wendt cohesin targets up | 0.751 | <0.001 |
| Kang doxorubicin resistance dn | 0.773 | 0.004 |
| Biocarta EGFR SMRTE pathway | 0.81 | 0.007 |
| Chr6q12 | 0.792 | 0.008 |
| Chr4q23 | 0.761 | 0.008 |
| Chr13q22 | 0.753 | 0.015 |
| Neurotransmitter secretion | 0.756 | 0.026 |
| Myllykangas amplification hot spot 7 | 0.833 | 0.027 |
| Chr3q11 | 0.752 | 0.029 |
| Mips TNF alpha NF kappa b signaling complex 10 | 0.779 | 0.038 |
| Th17 surface molecules | 0.764 | 0.039 |
| Vandesluis normal embryos up | 0.867 | 0.043 |

**Supplemental Figure 5:** Comparison between methods originally reported (Reinius) to updated estimateCellCounts2, Salas et al. (2018).

**Supplemental Table 2:** Diagnostic criteria for Panic Disorder With or Without Agoraphobia. DSM Version: IV

| Criteria For Panic Disorder With or Without Agoraphobia: |
| --- |
| 1. Both (1) and (2): |
| - 1. recurrent unexpected Panic Attacks |
| - 1. at least one of the attacks has been followed by 1 month (or more) of one (or more) of the following: |
| - - 1. persistent concern about having additional attacks |
| - - 1. worry about the implications of the attack or its consequences (e.g., losing control, having a heart attack, "going crazy") |
| - - 1. a significant change in behavior related to the attacks |
| 1. Presence or Absence of Agoraphobia. |
| 1. The Panic Attacks are not due to the direct physiological effects of a substance (e.g., a drug of abuse, a medication) or a general medical condition (e.g., hyperthyroidism). |
| 1. The Panic Attacks are not better accounted for by another mental disorder, such as Social Phobia (e.g., occurring on exposure to feared social situations),Specific Phobia (e.g., on exposure to a specific phobic situation), Obsessive-Compulsive Disorder (e.g., on exposure to dirt in someone with an obsession about contamination), Posttraumatic Stress Disorder (e.g., in response to stimuli associated with a severe stressor), or Separation Anxiety Disorder(e.g., in response to being away from home or close relatives). |

**Supplemental Figure 6:** Comparison between known and estimated cell-type proportions using the 1,426 leukocyte differentially methylated CpGs used for cell type deconvolution in this study.

**Supplemental Figure 7:** Comparison between age estimation methods. Blue is the Horvath age used in this study, while red and green are derived from Zhang et al. (2019) omic-data-based complex trait analysis (OSCA). Mean age from Best Linear Unbiased Prediction (BLUP) and Elastic Net methods were higher than using Horvath methods, which was closer to the mean reported by Iurato et al. (2017) (dashed line) and within a reported standard deviation (dotted line).
